# Acute Pancreatitis with Rapid Clinical Improvement in a Child with Isovaleric Acidemia

**DOI:** 10.1155/2013/721871

**Published:** 2013-02-04

**Authors:** Elpis Mantadakis, Ioannis Chrysafis, Emmanouela Tsouvala, Athanassios Evangeliou, Athanassios Chatzimichael

**Affiliations:** ^1^Department of Pediatrics, Democritus University of Thrace Faculty of Medicine, University General Hospital of Alexandroupolis, 68100 Alexandroupolis, Greece; ^2^Department of Radiology and Medical Imaging, University General Hospital of Alexandroupolis, 68100 Alexandroupolis, Greece; ^3^4th Department of Pediatric, Aristotle University of Thessaloniki School of Medicine, General Hospital Papageorgiou, 56429 Thessaloniki, Greece

## Abstract

Isovaleric acidemia is a rare branched-chain organic acidemia. The authors describe a 3.5-year-old girl with isovaleric acidemia and acute abdominal pain associated with bilious emesis. Elevated serum amylase and abdominal ultrasonography demonstrating an enlarged and edematous pancreas, along with the presence of peripancreatic exudates, confirmed the presence of acute pancreatitis. The patient recovered quickly with intravenous hydration, pancreatic rest, and administration of intravenous L-carnitine. Pancreatitis should be ruled out in the context of vomiting in any patient with isovaleric acidemia. Conversely, branched-chain organic acidemias should be included in the differential diagnosis of any child with pancreatitis of unknown origin.

## 1. Introduction

Acute pancreatitis is a life-threatening inflammatory condition with many known inciting factors, including inborn errors of metabolism [1–5]. One of them is isovaleric acidemia (aciduria), a branched-chain organic acidemia (BCOA) managed by restriction of protein intake to the amount needed for growth and by administration of nontoxic amino acids (glycine) and of L-carnitine [6].

We describe a young girl with isovaleric acidemia and acute pancreatitis and review its clinical and imaging findings and the relevant literature. 

## 2. Case Presentation

A 3.5-year-old girl was transferred to our institution from a local hospital with abdominal pain and bilious vomiting for further investigation and management. The child was diagnosed with isovaleric acidemia in the neonatal period, because there was a positive family history of a sibling death from this inherited disorder. Hence, measurement of elevated concentrations of isovalerylglycine in urine and of isovalerylcarnitine in plasma by mass spectrometry [7] was performed after birth; then, isovaleric acid CoA dehydrogenase deficiency was confirmed by assaying its enzymatic activity in cultured skin fibroblasts [8]. 

The child was admitted to the local hospital two days ago because of new-onset upper abdominal pain. Although she was unable to describe the nature and character of the pain, it was not severe enough to require analgesics. One day prior to her admission to us, she developed vomiting that eventually became bilious. 

The patient who had normal growth and development was on oral L-carnitine supplementation since infancy. Beside that, she was not receiving any other medications. Her family history was negative for pancreatitis. On admission to us, she was afebrile with normal vital signs. On physical examination, there was moderate upper abdominal tenderness without rebound, while she had normal bowel sounds and no organomegaly. Laboratory studies on admission revealed the following: leukocytes 10,110/*μ*L, hemoglobin 14 g/dL, hematocrit 37.2%, platelets 169,000/*μ*L, glucose 185 mg/dL (normal < 100 mg/dL), urea 26 mg/dL, creatinine 0.4 mg/dL, sodium 133 mEq/L, potassium 3.6 mEq/L, chloride 103 mEq/L, AST 34 U/L, ALT 22 U/L, LDH 426 U/L (normal < 280 U/L), albumin 4.3 g/dL, total bilirubin 0.7 mg/dL, direct bilirubin 0.2 mg/dL, *γ*-GT 16 U/L, triglycerides 80 mg/dL, calcium 10 mg/dL, and amylase 536 U/L (normal 40–140 U/L). Arterial blood gases showed the following: pH 7.55, pCO_2_ 21 mmHg, pO_2_ 133 mmHg, HCO_3_ 23 mmol/L, and base deficit 3.8 mmol/L. The anion gap was estimated at 7. A coagulation profile and a urine analysis were normal.

**Figure 1:**
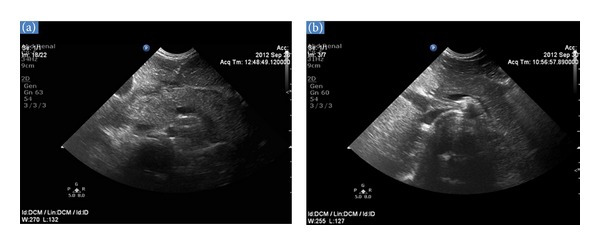
(a) Abdominal ultrasound on admission. Upper abdominal transverse section showing generalized enlargement of the pancreatic gland with a slightly heterogenous parenchyma and an overall reduction in reflectivity. Peripancreatic fluid collections are also noted. (b) Abdominal ultrasound on the 5th hospital day. Upper abdominal transverse section showing normalization of the size of the pancreas and complete resorption of the peripancreatic fluid collections.

Due to the bilious nature of vomiting, an abdominal ultrasound (Figure 1(a)) was obtained that showed a notably enlarged and edematous pancreas, along with the presence of peripancreatic exudates that were extending bilaterally towards the anterior paranephric spaces and towards the right colon. The wall of the duodenum was also diffusely edematous. No gallstones were seen, the bile duct was not distended, and there was no sludge in it, while the liver, spleen, and appendix were visualized without echomorphologic abnormalities. 

 Since the goal of management of acute pancreatitis is to achieve analgesia, adequate rehydration, and organ rest, the child was managed with discontinuation of oral feedings, placement of a nasogastric tube, intravenous hydration with 5% dextrose with electrolytes, and administration of intravenous ranitidine and of L-carnitine, 500 mg every 8 hours, that is, approximately 100 mg/kg/day. 

 The patient's clinical course was uncomplicated. Serum amylase normalized (84 U/L) on the 4th hospital day. She was fed with a low-fat and protein diet on the 6th hospital day. On the same day, the intravenous fluids were discontinued. Two follow-up ultrasonographic examinations on the 3rd and 5th (Figure 1(b)) hospital days showed progressive but rapid resolution of the pancreatic edema and of the peripancreatic exudates. Due to substantial clinical improvement, with complete disappearance of the abdominal pain, the child was discharged home without any complaints on the 8th hospital day. She continued to do well, two months after hospital discharge. 

## 3. Discussion

Isovaleric acidemia, also calledisovaleric aciduria, is a rare autosomal recessive disorder which disrupts the normal metabolism of the branched-chain amino acid leucine. It is due to isovaleric acid CoA dehydrogenase deficiency [9]. Along with methylmalonic and propionic acidemias, and maple syrup urine disease are the most common BCOAs, a group of metabolic diseases caused by enzyme deficiencies in the degradation of the branched-chain amino acids leucine, isoleucine, and valine. 

A characteristic presenting feature of isovaleric acidemia is the distinctive odor of sweaty feet during acute illness due to the buildup of isovaleric acidin affected individuals. In about 50% of the cases, signs and symptoms of this disorder become apparent within a few days after birth and include poor feeding, vomiting, seizures, and lethargythat can progress to coma and death [10]. In the other 50% of the cases, signs and symptoms of the disorder appear during childhood and may fluctuate over time. In these children, metabolic decompensation, which may result in acidosis, an increased anion gap, hyperammonemia, and ketonuria, is triggered by prolonged fasting, infections, or eating an increased amount of protein-rich foods. Metabolic decompensation is prevented by minimizing protein intake through avoidance of dairy products, eggs, meat, fish, legumes, and nuts [11]. Our patient did not develop metabolic acidosis and/or an increased anion gap likely due to the prompt institution of appropriate therapy, that is, intravenous hydration, pancreatic rest, and administration of intravenous L-carnitine.

Acute pancreatitis is a potentially life-threatening disorder that has many known inciting factors. Whereas many cases in children are idiopathic, among the well-established causes of acquired pancreatitis are trauma, biliary tract lesions, such as gallstones, viral or bacterial infections, medications, and systemic diseases such as hemolytic uremic syndrome (HUS). History ruled out trauma and medications as causes of pancreatitis, abdominal ultrasonography ruled out biliary tract pathology, and full blood count and biochemical studies ruled out HUS in our child.

Kahler et al. in 1994 were the first to associate acute pancreatitis with BCOAs. These authors described 9 children (7 with acute and 2 with chronic pancreatitis) among 108 children with BCOAs. The patients were 13-month to 9-year olds. Three had isovaleric acidemia, like our child. All the patients with isovaleric acidemia were identified after the occurrence of pancreatitis [11]. Although our patient had clinical, laboratory (elevated serum amylase), and imaging findings of acute pancreatitis, she was not acidotic and had a normal anion gap. 

Hypertriglyceridemia is a risk factor for pancreatitis, and elevations in free fatty acids sensitize the pancreas to pancreatitis [12]. In the glycogen storage disease type I, as in lipoprotein lipase deficiency, pancreatitis can be caused by a prominent elevation of serum triglycerides [13]. However, in isovaleric acidemia, the serum triglycerides are not usually elevated, and this was the case in our patient.

Patients with organic acidurias, aminoacidopathies, and congenital hyperammonemias often show gastrointestinal dysfunction including feeding refusal, nausea, vomiting, and gastroesophageal and abdominal pain during episodes of acute metabolic decompensation. In some cases, signs of gastrointestinal dysfunction may also be present, even when they are metabolically stable. Although it has been suggested that high-lipid diets in patients with organic acidemias may be associated with an increased risk for pancreatitis, inappropriate diet did not likely play a role in our patient's condition, since she followed meticulously the metabolic instructions given to her. 

The pathogenesis of pancreatitis in the organic acidemias is essentially unknown. Some of the proposed explanations are mitochondrial dysfunction resulting in lack of ATP, a direct effect on the pancreatic acinar cell membranes by accumulated toxic metabolites, deficiencies of carnitine, methionine, antioxidant agents, such as vitamin C, vitamin E, glutathione, and selenium, and increased free radicals [14, 15]. Since 50% of the patients with isovaleric acidemia may be asymptomatic in early life and pancreatitis may be one of the clinical manifestations, isovaleric acidemia should be included in the differential diagnosis of any child with pancreatitis of unknown origin.

In conclusion, acute pancreatitis can occur and should be ruled out in the context of vomiting in any patient with isovaleric acidemia. On the other hand, BCOAs should be contained within the differential diagnosis of any child with pancreatitis of unknown origin. Clinical and investigational studies are necessary to clarify the cause of pancreatitis in these patients.
